# Dietary fat quality and serum androgen concentrations in middle-aged men

**DOI:** 10.1038/s41430-023-01358-9

**Published:** 2023-10-27

**Authors:** Miika M. Wynne-Ellis, Jaakko J. Mursu, Tomi-Pekka Tuomainen, Elizabeth Bertone-Johnson, Jukka T. Salonen, Jyrki K. Virtanen

**Affiliations:** 1 University of Eastern Finland, Institute of Public Health and Clinical Nutrition, Kuopio, Finland; 2 University of Jyväskylä, Faculty of Sport and Health Sciences, Jyväskylä, Finland; 3 University of Massachusetts Amherst, Department of Health Promotion and Policy, Amherst, MA USA; 4 University of Helsinki, Faculty of Medicine, Department of Public Health, Helsinki, Finland; 5 Metabolic Analytical Services Oy, Helsinki, Finland

**Keywords:** Risk factors, Epidemiology

## Abstract

**Background/Objectives:**

Average testosterone concentrations in men have declined over the last few decades. The reasons for this are not fully known, but changes in dietary fat quality have been suggested to have a role. This study aimed to investigate the associations of different dietary fatty acids with serum androgen concentrations.

**Subjects/Methods:**

A total of 2546 men with a mean age of 53 from the Kuopio Ischaemic Heart Disease Risk Factor Study were included in this cross-sectional study. Associations between dietary saturated (SFA), monounsaturated (MUFA), polyunsaturated (PUFA) and trans (TFA) fatty acids and concentrations of serum total and free testosterone and steroid hormone binding globulin (SHBG) were analyzed with analysis of covariance and linear regression analysis. Associations of isocaloric replacement of nutrients and androgen concentrations were analyzed with multivariate nutrient-density models.

**Results:**

After adjustment for age, examination year and energy intake, higher SFA intake was associated with higher serum total and free testosterone and SHBG concentrations, and higher PUFA intake with lower concentrations. However, the associations were attenuated and not statistically significant after further adjustments for potential confounders. MUFA and TFA intakes were not associated with androgen concentrations. In isocaloric substitution models, replacing dietary protein with SFA was associated with higher serum total testosterone and SHBG concentrations. After excluding men with history of CVD or diabetes (*n* = 1021), no statistically significant associations were found.

**Conclusions:**

Dietary fat quality was not independently associated with serum androgen concentrations in middle-aged men. However, replacing protein with SFA may be associated with higher serum androgen concentrations.

## Introduction

Average testosterone concentrations have decreased at rates of up to 1% per year over the past 50 years, particularly in Western countries, [1, 2] and up to two in five men over 45 can be classified as having testosterone deficiency [3]. Testosterone has numerous implications for men’s health, as low testosterone concentrations have been associated with several morbidities, including sexual dysfunction [4], obesity [5], type 2 diabetes (T2DM) [6], and metabolic syndrome [7].

Testosterone concentrations are known to be affected at least by age [8], body mass index [9], alcohol consumption [10], smoking [11] and physical activity [12, 13]. Besides these factors, at the same time as testosterone concentrations have been decreasing, there has been a major shift in dietary habits. It is proposed that some of the decreases in testosterone concentrations in the Western populations could be due to changes in diet. [14]. Recent studies suggest that as intake of SFA has remained stable and the intake of MUFA and especially intake of PUFA has increased in the USA and Europe, one possible contributor to lower testosterone levels could be the increase in intake of PUFA [15, 16]. Although connections between dietary fat intake, especially intake of SFA, MUFA and PUFA, and androgen concentrations have been observed [17–19], other studies on the association between fatty acid intake and androgen concentrations have produced conflicting results [20–23].

However, there is a paucity of research data on the impact of individual dietary fatty acids on androgen concentrations in men, although there is some evidence that different dietary fats could affect testosterone concentrations. In animal models [24, 25], SFA has been linked to positive and PUFA, especially omega-6 PUFA, to negative effects on concentrations of steroidogenic enzymes and the function of testes. Findings could indicate that there could be an effect of individual fatty acids on androgen concentrations [24, 25]. In a few experimental studies on men [26, 27], findings indicate that especially intake of PUFA and MUFA could have negative effects on men’s androgen concentrations, although other experimental studies found no connections [28, 29].

As the number of studies is limited and findings conflicting on the associations of different dietary fatty acids with androgen concentrations in humans, we aimed to investigate the associations of dietary fatty acids with serum total testosterone, free testosterone and SHBG among middle-aged men from Eastern Finland.

## Subjects and Methods

### Study population

The Kuopio Ischaemic Heart Disease Risk Factor Study (KIHD) is an ongoing population-based study designed to investigate risk factors for cardiovascular diseases (CVD) and other chronic diseases in middle-aged men and women from eastern Finland. The study protocol was approved by the Research Ethics Committee of the University of Kuopio (approval number: 01/12/1983). All subjects gave their informed consent.

The data used for the current analysis was collected at the baseline examinations of the KIHD in 1984–1989 from a total of 2682 men who were 42, 48, 54, or 60 years old at baseline and living in the city of Kuopio or the neighbouring communities.

From the current analyses we excluded participants who had data missing on serum androgen concentrations (*n* = 59) or on dietary intakes (*n* = 26). In addition, we excluded participants with cancer (*n* = 50) and those on hormone therapy (*n* = 1). The final number of men in the main analyses was 2546. In the additional analyses we excluded men with history of CVD or diabetes (*n* = 1021), because these diseases are associated with lower serum androgen concentrations [30, 31].

### Measurements

The subjects gave fasting blood samples between 8 and 10 AM. They were instructed to abstain from ingesting alcohol for three days and from smoking and eating for 12 h before giving the sample. Detailed descriptions of the determination of serum lipids and lipoproteins, assessment of medical history and medications, family history of diseases, smoking, alcohol consumption and blood pressure have been published by Salonen et al. [32]. Education was assessed in years by using a self-administered questionnaire. Annual income was obtained from a self-administered questionnaire. Diabetes was defined as self-reported diabetes mellitus or fasting blood glucose of ≥ 6.7 mmol/L. Physical activity was assessed using the KIHD 12-Month Leisure-Time Physical Activity Questionnaire which covers the type, frequency, duration and intensity of the activity [33]. Body mass index (BMI) was computed as the ratio of weight in kilograms to the square of height in metres, both measured by a trained nurse at study visit.

### Measurement of sex hormones and SHBG

Sex hormone-binding globulin (SHBG) was determined using a 1235 AutoDELFIA automatic system based on a time-resolved fluoroimmunoassay (AutoDELFIA SHBG, Wallac Co., Turku, Finland). Total testosterone levels were measured with the AutoDELFIA Testosterone kit (Wallac Co.). Non-SHBG-bound, free testosterone (fT) was calculated using the following formula: proportion (%) of fT (fT%) = 2.28–1.38*log(SHBG nmol/1/10), and serum fT (pmol/l) = fT%*T (nmol/l)*10 [34].

### Dietary assessment

Dietary intakes were assessed using a 4-day food record of three weekdays and one weekend day at the time of the baseline examinations. Participants received instructions from a nutritionist on how to complete the food records using conventional household measures. To aid with portion-size estimates, participants received a book with pictures of 126 common foods and dishes. The book contained pictures of 3–5 commonly used portion sizes for each food item. The participant could also describe the portion size with the examples in the book. The completed food records were cross-checked by a nutritionist together with a participant to minimize reporting bias.

Food and nutrient intakes were estimated as the mean intake over the four days from the food records using the NUTRICA® 2.5 software (Social Insurance Institution, Turku, Finland). The databank of the software is mainly based on Finnish values of the nutrient composition of foods.

### Statistical analyses

ANCOVA and linear regression were used for analyses. Equality of variances and normality of the variable distributions were confirmed. The analyses were controlled for potential confounders, which are known to affect androgen concentrations. Model 1 included age (years), examination year and energy intake (kcal/day). Model 2 was adjusted for Model 1 plus alcohol intake (g/day), pack-years of smoking, BMI (kg/m^2^), years of education, physical activity (kcal/day), history of CVD or diabetes as one variable (yes/no), and fruit, berry, and vegetable intake (g/day). The coefficient from these models for a specific fatty acid is interpreted as replacing energy coming from the specific fatty acid with equivalent energy coming from all other sources of energy, i.e., protein, carbohydrates and the other fatty acids.

The associations with serum androgen concentrations of replacing a specific energy source with another were investigated by isocaloric substitution, using multivariable (Model 2) nutrient-density models [35, 36]. For these analyses, a 2 percent of total daily energy intake (E%) change in the intake of an energy source was used. For example, when investigating the associations of substituting energy coming from protein for equivalent energy coming from the different fatty acids, the model included the covariates (see the list above) and intakes of carbohydrates, SFA, PUFA, trans fatty acids (TFA) and monounsaturated fatty acids (MUFA). When investigating the substitution of one fatty acid for another, the model included the covariates (listed above) and protein, carbohydrates and three out of four fatty acids. For example, when investigating the substitution of SFA (or TFA or MUFA) for PUFA, the model included protein, carbohydrates, SFA, TFA and MUFA but not PUFA [36]. The statistical significance of the linear trend across the quartiles was analyzed by assigning the median values for each category of exposure variable and treating those as a single continuous variable. All *p*-values were 2-tailed (*α* = 0.05). SPSS 26 for Windows (Armonk, NY: IBM Corp.) was used for analyzing the data.

## Results

### Baseline characteristics

Baseline characteristics for the entire study population are presented in Table 1 and according to the quartile of fatty acid intakes in the supplemental Table 1. Those with higher SFA intake had lower intake of PUFA, higher intake of MUFA and lower intake of fruits, berries and vegetables. Higher intake of SFA was also associated with fewer years of education and lower leisure-time physical activity. Higher intake of PUFA was associated with lower SFA intake, higher MUFA intake and higher intake of fruits, berries and vegetables. Those with higher PUFA intake also had more years of education and higher leisure-time physical activity and were less likely to be smokers than those with lower PUFA intake. Subjects with a higher intake of MUFA had a higher intake of SFA and PUFA but a lower intake of fibre and fruits, berries and vegetables. Subjects with a higher intake of MUFA had also less leisure time physical activity than those with a lower intake of MUFA. TFA was not clearly associated to other factors, possibly due to the low overall intake of TFA.

**Table 1:** Characteristics of the 1981 men in the Kuopio Ischaemic Heart Disease Risk Factor Study (KIHD) in 1984–1989.

**Characteristic**	
Age, y	52.5 ± 5.3
Education, y	9.0 ± 3.6
Leisure-time physical activity, kcal/d	138 ± 168
Body mass index, kg/m2	26.7 ± 3.5
Alcohol intake, g/wk	73 ± 115
Current smoker, %	30
Diabetes, %	5
Family history of coronary heart disease, %	46
**Dietary intakes**	
Energy, kcal/d	2391 ± 628
Total fat, E%	38.9 ± 6.3
Saturated fat, E%	18.0 ± 4.1
Trans fat, E%	1.1 ± 0.4
Monounsaturated fat, E%	11.0 ± 2.2
Polyunsaturated fat, E%	4.6 ± 1.5
Protein, E%	15.6 ± 2.7
Carbohydrates, E%	43.5 ± 6.6
Fibre, g/d	25 ± 9

*E%* Per cent of energy intake.

### Dietary fatty acid intake and serum total and free testosterone concentrations

Higher SFA intake was associated with higher serum total and free testosterone concentrations in Model 1, which was adjusted for age, year of study and energy intake (Tables 2 & 3). The difference in the serum total testosterone concentration between the highest and the lowest quartile of SFA intake was 1.7 nmol/L [95% confidence interval (CI) 0.8–2.6 nmol/L, P for trend across the quartiles < 0.001] and in free testosterone concentrations 11.7 pmol/L (95% CI 2.6–20.8 pmol/L, P-trend 0.004). After further adjustments for potential confounders (Model 2, Tables 2 & 3) the associations were lost.

**Table 2:** The mean concentrations of serum total testosterone (nmol/L) in quartiles of dietary fatty acid intakes in the 2546 middle-aged KIHD men.

	Intake quartile				
	1 (*n* = 636)	2 (*n* = 637)	3 (*n* = 637)	4 (*n* = 636)	P-trend
**Saturated fatty acids**					
Median intake (E%)	13.6	16.7	19.4	22.9	
Model 1	20.5 ± 0.3	20.3 ± 0.3	21.0 ± 0.3	22.2 ± 0.3	<0.001
Model 2	21.1 ± 0.3	20.5 ± 0.3	20.8 ± 0.3	21.5 ± 0.3	0.296
**Trans fatty acids**					
Median intake (E%)	0.7	0.9	1.1	1.5	
Model 1	21.0 ± 0.3	20.7 ± 0.3	21.4 ± 0.3	21.0 ± 0.3	0.824
Model 2	21.5 ± 0.3	20.6 ± 0.3	21.0 ± 0.3	20.9 ± 0.3	0.286
**Monounsaturated fatty acids**					
Median intake (E%)	9.3	10.1	12.2	14.2	
Model 1	20.4 ± 0.3	21.8 ± 0.3	21.0 ± 0.3	20.8 ± 0.3	0.881
Model 2	20.6 ± 0.3	21.6 ± 0.3	20.9 ± 0.3	20.9 ± 0.3	0.922
**Polyunsaturated fatty acids**					
Median intake (E%)	2.0	3.9	4.8	6.2	
Model 1	21.7 ± 0.3	21.1 ± 0.3	21.0 ± 0.3	20.2 ± 0.3	<0.001
Model 2	21.3 ± 0.3	20.8 ± 0.3	21.4 ± 0.3	20.6 ± 0.3	0.215

Values are means ± SEM.

Model 1 is adjusted for age, examination year and energy intake.

Model 2 is adjusted for Model 1, plus alcohol intake, smoking, BMI, years of education, physical activity, history of cardiovascular disease or diabetes, and intake of fruits, berries and vegetables.

**Table 3:** The mean serum concentrations of free testosterone (pmol/L) in quartiles of dietary fatty acid intakes in the 2546 middle-aged KIHD men.

	Intake quartile				
	1 (*n* = 636)	2 (*n* = 637)	3 (*n* = 637)	4 (*n* = 636)	P-trend
**Saturated fatty acids**					
Median intake (E%)	13.6	16.7	19.4	22.9	
Model 1	296 ± 3.2	294.7 ± 3.1	301.6 ± 3.1	307.7 ± 3.2	0.004
Model 2	300.8 ± 3.2	296.7 ± 3.0	300.2 ± 3.0	302.2 ± 3.3	0.634
**Trans fatty acids**					
Median intake (E%)	0.7	0.9	1.1	1.5	
Model 1	300.0 ± 3.1	296.8 ± 3.1	302 ± 3.1	301.2 ± 3.1	0.558
Model 2	304.0 ± 3.1	296.3 ± 3.0	299.3 ± 3.0	300.4 ± 3.0	0.644
**Monounsaturated fatty acids**					
Median intake (E%)	9.3	10.1	12.2	14.2	
Model 1	295.2 ± 3.1	305.9 ± 3.1	299.4 ± 3.1	299.5 ± 3.1	0.629
Model 2	297.2 ± 3.0	304.4 ±3.08	298.3 ± 3.0	300.0 ± 3.0	0.862
**Polyunsaturated fatty acids**					
Median intake (E%)	2.0	3.9	4.8	6.2	
Model 1	305.1 ± 3.1	300.8 ± 3.1	300.1 ± 3.1	294.1 ± 3.2	0.018
Model 2	301.8 ± 3.1	298.6 ± 3.0	302.6 ± 3.0	297 ± 3.1	0.393

Values are means ± SEM.

Model 1 is adjusted for age, examination year and energy intake.

Model 2 is adjusted for Model 1, alcohol, smoking, BMI, years of education, physical activity, history of cardiovascular disease or diabetes and intake of fruits, berries and vegetables.

Higher dietary PUFA intake was associated with lower serum total and free testosterone concentrations in Model 1. The difference in the highest vs. the lowest quartile was 1.6 nmol/L (95% CI 0.7–2.4 nmol/L, P-trend < 0.001) in the serum total testosterone concentrations and 11.0 pmol/L (95% CI 2.1–19.9 pmol/L, P-trend = 0.018) in the serum free testosterone. However, the associations were attenuated and not statistically significant after further adjustments for potential confounders (Model 2, Tables 2 & 3). MUFA and TFA intakes were not associated with serum testosterone concentrations in either of the models.

### Dietary fatty acid intake and serum SHBG concentrations

Higher SFA intake was associated with higher serum SHBG concentrations and higher PUFA intake with lower concentrations in model 1 (Table 4). The difference in the serum SHBG concentration between the highest and the lowest quartile of SFA intake was 3.9 nmol/L (95% CI 1.9–5.9 nmol/L, P-trend < 0.001) and in the highest vs. the lowest PUFA intake quartile 3.9 nmol/L (95% CI 2.0–5.8 nmol/L, P-trend < 0.001). The associations were again attenuated and not statistically significant after further adjustments for potential confounders (Model 2, Table 4). MUFA and TFA intakes were not associated with serum SHBG concentrations.

**Table 4:** The mean serum concentrations of SHBG (nmol/L) in quartiles of dietary fatty acid intakes in the 2546 middle-aged KIHD men.

	Intake quartile				
	1 (*n* = 636)	2 (*n* = 637)	3 (*n* = 637)	4 (*n* = 636)	P-trend
**Saturated fatty acids**					
Median intake (E%)	13.6	16.7	19.4	22.9	
Model 1	39.5 ± 0.7	38.6 ± 0.7	40.2 ± 0.7	43.4 ± 0.7	<0.001
Model 2	40.7 ± 0.7	39.2 ± 0.6	39.9 ± 0.6	41.8 ± 0.7	0.225
**Trans fatty acids**					
Median intake (E%)	0.7	0.9	1.1	1.5	
Model 1	40.3 ± 0.7	40.0 ± 0.7	41.4 ± 0.7	39.8 ± 0.7	0.793
Model 2	41.3 ± 0.6	39.9 ± 0.6	40.6 ± 0.6	39.8 ± 0.6	0.187
**Monounsaturated fatty acids**					
Median intake (E%)	9.3	10.1	12.2	14.2	
Model 1	39.6 ± 0.7	42.3 ± 0.7	40.5 ± 0.7	39.2 ± 0.7	0.315
Model 2	40.0 ± 0.6	41.9 ± 0.6	40.2 ± 0.6	39.6 ± 0.6	0.28
**Polyunsaturated fatty acids**					
Median intake (E%)	2.0	3.9	4.8	6.2	
Model 1	42.3 ± 0.7	40.6 ± 0.7	40.2 ± 0.7	38.4 ± 0.7	<0.001
Model 2	41.2 ± 0.6	39.9 ± 0.6	41.1 ± 0.6	39.5 ± 0.6	0.135

Values are means ± SEM.

Model 1 is adjusted for age, examination year and energy intake.

Model 2 is adjusted for Model 1, alcohol, smoking, BMI, years of education, physical activity, history of cardiovascular disease or diabetes and intake of fruits, berries and vegetables.

### Isocaloric substitution analyses

**Fig. 1:**
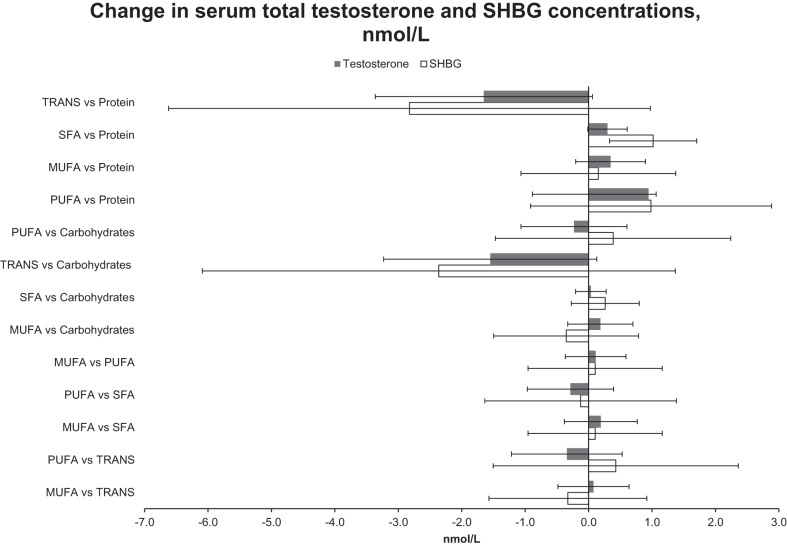
The differences in concentrations of serum total testosterone and sex hormone-binding globulin (SHBG) with multivariate-adjusted isocaloric substitution of 2 percent of total energy intake (E%) from one dietary component for another. Model adjusted for age, examination year, energy intake, alcohol, smoking, body mass index), years of education, physical activity, history of cardiovascular disease or diabetes, and intake of fruits, berries and vegetables.

**Fig. 2:**
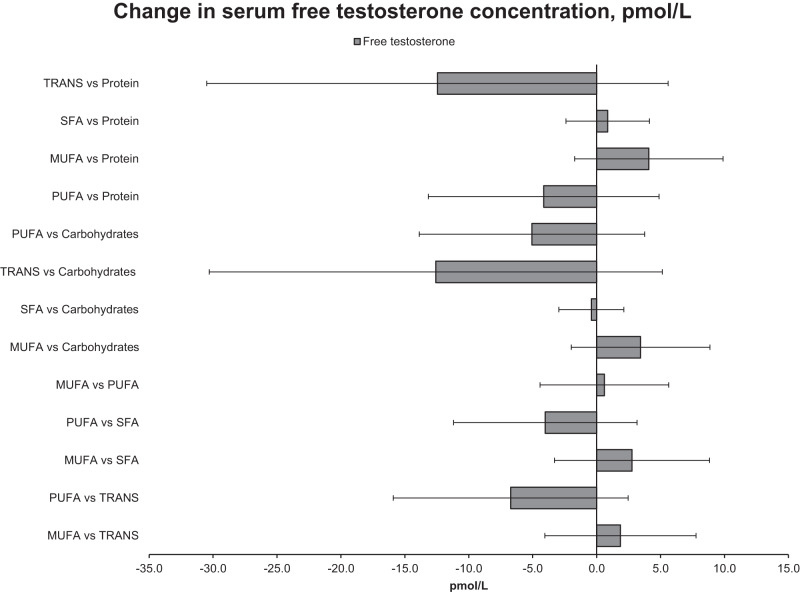
The difference in concentration of serum free testosterone with multivariate-adjusted isocaloric substitution of 2 percent of total energy intake (E%) from one dietary component for another. Model adjusted for age, examination year, energy intake, alcohol, smoking, body mass index, years of education, physical activity, history of cardiovascular disease or diabetes, and intake of fruits, berries and vegetables.

Figures 1 and 2 show the difference in serum androgen concentrations with multivariate-adjusted isocaloric substitution of 2 E% from one dietary component for another. The only statistically significant association was observed with higher SFA intake in place of protein. Replacing energy coming from protein with energy from SFA was associated with 0.3 nmol/L (95% CI −0.01 to 0.6 nmol/L) higher serum total testosterone and 1.0 nmol/L (95% CI 0.3–1.7 nmol/L) higher SHBG concentrations (Fig. 1).

### Additional analyses

The associations between fatty acid intakes and serum testosterone, free testosterone and SHBG concentrations were generally similar when energy-adjusted fatty acid intakes in grams per day were used in the analyses (Supplemental Tables 2–4).

After restricting the analyses to men without CVD or diabetes (*n* = 1525), no statistically significant associations were found in the categorical analyses or in the isocaloric substitution analyses. For example, in the multivariable-adjusted isocaloric substitution analyses (model 2) the results that replacing energy coming from protein with energy from SFA increasing testosterone concentrations 0.3 nmol/L (CI −0.01 to 0.6) were attenuated, and testosterone concentrations were found to increase 0.13 nmol/L (CI −0.28 to 0.54). Similarly, the observed rise of 1.0 nmol/L (CI 0.3–1.7) in SHBG, when energy from protein was replaced with energy from SFA was attenuated to 0.73 nmol/L (CI −0.19 to 1.66) (other data not shown).

## Discussion

In this population-based cohort study in Finnish middle-aged men, dietary intakes of SFA, PUFA, MUFA or TFA were not independently associated with concentrations of serum total testosterone, free testosterone or SHBG after adjustment for potential confounders. In the isocaloric substitution model, higher SFA intake was associated with slightly higher serum total testosterone and SHBG concentrations when compared to the energy coming from protein. However, these associations were not observed in the analyses restricted to men without CVD or diabetes.

Differing from our study, most of the observational studies have found associations between dietary fatty acid intake and men’s androgen concentrations. In a study of 12 strength-trained men with a mean age of 27 years, a higher intake of PUFA was associated with lower testosterone concentrations and higher intake of SFA and MUFA with higher testosterone concentrations [19]. Similar findings were also found in a study of 69 men aged 43–88 years, in whom higher intake of PUFA was associated with lower concentrations of testosterone [22]. Contrary findings have been also reported. In a study of 108 men with a mean age of 41 years higher PUFA intake was related to higher testosterone concentrations [37]. In another study of 209 men aged 18–23 years, higher intakes of MUFA and TFA were associated with lower testosterone concentration [23]. In line with our findings, a study of 1317 men with a mean age of 44 years found no significant associations between dietary fatty acid intake and androgen concentrations [38]. Similarly, in a study of 636 men with mean age of 61 years, no associations between dietary fatty acid intake and androgen concentrations were found [39]. A large cross-sectional study of 3128 men compared a low-fat diet and a Mediterranean diet high in MUFA and PUFA with a nonrestricted diet and found that men on a low-fat and Mediterranean diets had modestly lower serum testosterone levels [20].

To this day only few experimental studies have investigated the effects of different fatty acids on men’s androgen concentrations. One pilot study found that an intake of PUFA from 50 grams of soybean oil decreased serum testosterone concentrations by 3.2 nmol/L after one hour compared to baseline, with this suppression remaining significant up to five hours postprandially [26]. However, increased dietary intake of arachidonic acid from arachidonic acid supplementation in men did not influence serum testosterone concentrations compared to control group ingesting corn oil (linoleic acid) supplementation in a randomised placebo controlled double-blinded study [40]. In a cross-over design study consisting of 13 men with hypertension and 13 non-hypertensive men, a dose of 5 g of long-chain n-3 PUFA (EPA + DHA) did not affect testosterone concentrations after 30 days [28]. Similarly in a double-blind, placebo-controlled trial of low-dose (400 mg/day) supplementation of n-3 PUFA, 1850 male post-myocardial infarction patients aged 60–80 years experienced no change in their testosterone concentrations [29]. Besides PUFA, increased intake of MUFA has also been shown to increase testosterone concentration in a study where 25 g of butter in the diet was replaced with olive oil or argan oil [27]. However, intake of MUFA has also been observed to decrease post-prandial testosterone concentrations after the consumption of 55 grams of MUFA from extra virgin olive oil [26].

Experimental studies on high-fat and low-fat diets have found associations of fat intake with testosterone concentrations, but the results are partly contradictory, as are observational studies. One study found that switching from a high-fat (40E%) to a low-fat (25E%) diet reduced testosterone concentrations in 35 Finnish middle-aged men [41]. Similarly, 43 American men aged 19–56 years on a high-fat (40E%) diet were found to have higher testosterone concentrations than those on a low-fat (20E%) diet [42]. A study with 39 middle-aged men also found that 8 weeks on a low-fat (14E%) diet lowered testosterone concentrations [43]. On the other hand, a recent study of 55 men aged 19-35 years showed no changes in androgen concentrations after a 12-week dietary intervention in which half followed a high-fat (40E%) and half a low-fat (22E%) diet [44]. Overall, the findings from observational and experimental studies on the associations between dietary fatty acids and androgen concentrations in men are conflicting. To date, none of the observational studies have used an isocaloric substitution model to investigate the relationship between testosterone concentrations in men and dietary fatty acids.

Although different fatty acids were not independently associated with serum androgen concentrations in our study, in isocaloric substitution analyses SFA intake was associated with slightly higher serum total testosterone and SHBG concentrations when replacing energy coming from protein. In several observational studies, higher protein intake has been associated with lower testosterone concentrations [19, 38, 45], whereas in experimental studies dietary protein intake does not appear to have a major effect on testosterone concentrations [46, 47]. Higher SFA intake can affect serum androgen concentrations due to fatty acid’s capability of binding androgens by plasma proteins [48], making them regulators of androgen bioavailability for tissues [49]. In this study cohort as in many other cohorts from Western countries, protein and SFA have many of the same sources, such as dairy and meat products, which makes it difficult to interpret and thus generalize the observed results of the substitution analyses. Also, these associations were not observed in the analyses restricted to men without CVD or diabetes, so the results should not be over-generalized. Besides the lower number of participants in the analyses, there is no apparent explanation for the attenuated associations among men with history of these diseases, as there were no marked differences in the fatty acid intakes compared to the whole study population (data not shown). However, our analysis was cross-sectional, so it is difficult to disentangle potentially causal effects from the effects of CVD or diabetes treatment on androgen levels, and/or treatment related changes in fat intake on the association of fat intake with androgen concentrations. Finally, because the difference in testosterone and SHBG concentrations were small, the clinical significance of the finding is not clear.

This study has multiple strengths including population-based recruitment, detailed information on dietary intakes and the large number of potential confounders that could be taken into account in statistical analyses. Dietary intakes were assessed with a four-day food recording, which is considered the gold standard in assessing dietary intakes in population studies. Limitations of this study include the cross-sectional and observational design, which makes causal interpretations difficult. Also, serum androgen concentrations were measured only once, although there is known heterogeneity in serum testosterone concentrations from the same patient, and therefore, multiple measurements would have decreased random error and increased precision [50]. The study population consists of middle-aged Caucasian men located in a geographically exclusive area, limiting the generalizability of the results.

In conclusion, our results suggest that the quality of dietary fat is not independently associated with serum androgen concentrations in middle-aged men from eastern Finland, although higher SFA intake in place of protein was associated with slightly higher serum-free and total testosterone concentrations. Reduced testosterone concentrations are known to have several adverse effects on male health, but currently, there is little evidence of the impact of dietary factors on serum androgen concentrations. There is a need for well-designed, sufficiently powered, randomized controlled trials with strict dietary control to elucidate the role of dietary fatty acids on serum androgen concentrations. However, the costs of such trials can be high and weaknesses of RCTs, such as diminishing compliance in long trials and difficulty in blinding when changes in food intake are needed, can affect the results. Therefore, well-controlled observational studies that would especially take into consideration the aspect of replacement nutrient(s) can also provide important information.

### Supplementary information

Supplemental tables

## Data Availability

The data will not be openly available because it contains sensitive personal information of the participants that cannot be completely anonymized.
