# Supplementary material for: Dietary fat quality and serum androgen concentrations in middle-aged men
Source: Eur J Clin Nutr. 2023 Oct 27;78(2):99–106. doi: 10.1038/s41430-023-01358-9 (PMC10853065; doi:10.1038/s41430-023-01358-9)
Supplement: Supplementary file 1 — Supplemental tables [file 41430_2023_1358_MOESM1_ESM.docx]

| Supplemental Table 1. Baseline characteristics according to dietary saturated, monounsaturated, polyunsaturated and trans fatty acid intakes in men in the Kuopio Ischaemic Heart Disease Risk Factor Study (KIHD) in 1984-1989. | | | | | | | | | | | | | | |
| --- | --- | --- | --- | --- | --- | --- | --- | --- | --- | --- | --- | --- | --- | --- |
|  | | Quartile of saturated fat intake | | | | | |  |  |  | Quartile of trans fat intake | | |  |
|  | | 1  (n=636) | | 2  (n=637) | | 3  (n=637) | | 4  (n=636) | |  | 1  (n=636) | 2  (n=637) | 3  (n=637) | 4  (n=636) |
| Age, y | | 53.3±5.5 | | 52.3±5.2 | | 52.7±5.2 | | 53.8±4.3* | |  | 52.5±5.6 | 52.9±5.2 | 53.4±4.6 | 53.3±5.1* |
| Education, y | | 9.2±3.7 | | 9.24±3.7 | | 8.74±3.5 | | 7.4±2.4* | |  | 8.8±3.3 | 8.6±3.5 | 8.4±3.3 | 8.8±3.6 |
| Leisure-time physical activity, kcal/d | | 174±197 | | 155±174 | | 130±157 | | 104±158* | |  | 153±167 | 135±177 | 136±193 | 140±158 |
| Body mass index, kg/m^2^ | | 27.3±3.8 | | 26.9±3.4 | | 26.7±3.5 | | 26.7±3.6* | |  | 27.4±3.7 | 26.8±3.6 | 26.6±3.5 | 26.8±3.4* |
| Current smoker, % | | 18 | | 19 | | 25 | | 38* | |  | 22 | 25 | 30 | 25 |
| Alcohol intake, g/wk | | 101±207 | | 75±112 | | 68±97 | | 55±85* | |  | 112±209 | 74±113 | 59±97 | 53±72* |
| Diabetes, % | | 7 | | 5 | | 6 | | 6 | |  | 6 | 5 | 5 | 7 |
| Family history of coronary heart disease, % | | 40 | | 36 | | 33 | | 41 | |  | 34 | 35 | 39 | 42* |
| *Dietary intakes* | |  | |  | |  | |  | |  |  |  |  |  |
| Energy, kcal/d | | 2213±568 | | 2368±536 | | 2512±586 | | 2666±678* | |  | 2317±606 | 2484±601 | 2527±646 | 2432±597* |
| Total fat, E% | | 32.2±4.4 | | 37.3±3.4 | | 40.3±3.3 | | 45±3.9* | |  | 35.4±6.1 | 37.8±4.9 | 40.4±4.9 | 41±6.2* |
| Saturated fat, E% | | 13.2±1.8 | | 16.7±0.7 | | 19.4±0.8 | | 23.5±2.3* | |  | 15.6±3.2 | 18.2±2.9 | 20.1±3.7 | 18.8±5* |
| Trans fat, E% | | 1±0.4 | | 1±0.4 | | 1±0.4 | | 1.2±0.2* | |  | 0.7±0.1 | 0.9±0.1 | 1.1±0.6 | 1.6±0.3* |
| Monounsaturated fat, E% | | 10.7±2.2 | | 11.8±2.2 | | 12±2.2 | | 12.3±2* | |  | 11.1±2.3 | 11.1±2 | 11.5±1.7 | 13.1±2.2* |
| Polyunsaturated fat, E% | | 5.1±1.4 | | 4.9±1.5 | | 4.4±1.3 | | 3.7±1.1* | |  | 4.7±1.3 | 4.1±1.1 | 4±1.3 | 5.2±1.6* |
| Protein, E% | | 16.6±2.9 | | 16.1±2.4 | | 15.6±2.3 | | 14.7±2.1* | |  | 16.4±2.8 | 15.9±2.6 | 15.2±2.1 | 15.5±2.5* |
| Carbohydrates, E% | | 47.3±6.6 | | 43.7±5.6 | | 41.5±5.2 | | 38.4±5* | |  | 44±7.4 | 43.4±6 | 42.2±5.8 | 41.4±6.4* |
| Fiber, g/d | | 26.7±9.9 | | 24.5±8 | | 25±8.1 | | 24.1±8.6* | |  | 25.4±9.8 | 25.5±8.5 | 24.8±8.1 | 24.6±8.7 |
| Fruits, berries and vegetables, g/d^†^ | | 310±183 | | 275±149 | | 240±138 | | 181±123* | |  | 279±177 | 252±147 | 237±145 | 238±155* |
|  | |  | |  | |  | |  | |  |  |  |  |  |
|  | | Quartile of monounsaturated fat intake | | | | | | | |  | Quartile of polyunsaturated fat intake | | | |
|  | | 1  (n=636) | | 2  (n=637) | | 3  (n=637) | | 4  (n=636) | | | 1  (n=636) | 2  (n=637) | 3  (n=637) | 4  (n=636) |
| Age, y | | 53.9±4.6 | | 53.4±4.9 | | 52.9±5.2 | | 51.9±5.5* | |  | 54±4.1 | 53.4±4.6 | 52.9±5.3 | 51.7±5.9* |
| Education, y | | 8.3±3.4 | | 8.6±3.4 | | 8.7±3.6 | | 9±3.4* | |  | 7.44±2.5 | 8.3±3.2 | 9.1±3.6 | 9.7±3.9* |
| Leisure-time physical activity, kcal/d | | 152±200 | | 144±174 | | 133±168 | | 135±151 | |  | 120±178 | 145±180 | 145±179 | 154±157* |
| Body mass index, kg/m^2^ | | 27±3.6 | | 26.6±3.8 | | 26.8±3.3 | | 27.1±3.5 | |  | 26.8±3.7 | 26.6±3.5 | 27.1±3.4 | 27.1±3.6* |
| Current smoker, % | | 19 | | 28 | | 27 | | 25* | |  | 31 | 29 | 21 | 20* |
| Alcohol intake, g/wk | | 92±213 | | 67±99 | | 67±100 | | 72.4±88* | |  | 82±197 | 75±120 | 70±107 | 72±93 |
| Diabetes, % | | 4 | | 5 | | 5 | | 8* | |  | 5 | 5 | 7 | 7 |
| Family history of coronary heart disease, % | | 38 | | 36 | | 38 | | 38 | |  | 39 | 36 | 38 | 38 |
| *Dietary intakes* | |  | |  | |  | |  | |  |  |  |  |  |
| Energy, kcal/d | | 2384±644 | | 2466±632 | | 2481±585 | | 2429±604 | |  | 2580±678 | 2487±662 | 2371±538 | 2321±547* |
| Total fat, E% | | 32.9±4.8 | | 37.7±4.1 | | 40.2±4.5 | | 43.8±4.5* | |  | 38.6±6.4 | 38.2±6.2 | 38±5.5 | 39.9±5.5* |
| Saturated fat, E% | | 16.2±3.9 | | 18.4±3.8 | | 18.9±4.1 | | 19.3±3.9* | |  | 20.6±4.3 | 18.7±4.1 | 17.2±3.4 | 16.3±3.1* |
| Trans fat, E% | | 0.9±0.2 | | 1±0.3 | | 1.1±0.3 | | 1.3±0.5* | |  | 1±0.2 | 1±0.2 | 1±0.3 | 1.3±0.5* |
| Monounsaturated fat, E% | | 9±0.9 | | 10.9±0.4 | | 12.3±0.4 | | 14.6±1.4* | |  | 10±1.5 | 11.1±1.6 | 12±1.7 | 13.7±2.2* |
| Polyunsaturated fat, E% | | 3.5±0.9 | | 4±1 | | 4.6±1.1 | | 5.8±1.4* | |  | 2.9±0.4 | 3.9±0.2 | 4.8±0.3 | 6.4±1* |
| Protein, E% | | 15.9±2.9 | | 15.6±2.5 | | 15.7±2.3 | | 15.9±2.4 | |  | 15±2.4 | 15.6±2.4 | 16.1±2.5 | 16.3±2.6* |
| Carbohydrates, E% | | 47.9±6.2 | | 44.1±4.9 | | 41.4±4.9 | | 37.6±5.2* | |  | 43.5±6.3 | 43.3±6.8 | 43.2±6.2 | 40.1±6.4* |
| Fiber g/d | | 27.8±9.9 | | 25.8±8.9 | | 24.4±7.5 | | 22.3±7.6* | |  | 25.8±9.1 | 25.1±8.3 | 25.3±9.4 | 24.2±8* |
| Fruits, berries and vegetables, g/d^†^ | | 288.8±178.8 | | 253±143 | | 244±150 | | 220±146* | |  | 217±148 | 248±153 | 270±157 | 271±164.7* |
| E%, percent of energy intake  *p <0.05 for the trend across the fatty acid quartiles  ^†^Excluding potatoes | | | | | | | | | | | | | | |
| Supplemental Table 2. The mean serum concentrations of free testosterone (pmol/L) in quartiles of energy-adjusted dietary fatty acid intakes among middle-aged and older men | | | | | | | | | | | | |  |  |
|  | | Intake quartile | | | | | | | | | |  |  |  |
|  | | 1 (n=636) | | 2 (n=637) | | 3 (n=637) | | 4 (n=636) | | | | P-trend |  |  |
| Saturated fatty acids | | | | | |  | |  | | | |  |  |  |
| Median intake (g/d) | | 38.0 | | 45.9 | | 52.2 | | 61.4 | | | |  |  |  |
| Model 1 | | 293.8±6.4 | | 298.9±6 | | 299.3±6 | | 307.9±6.4 | | | | 0.002 |  |  |
| Model 2 | | 297.2±6.4 | | 301.8±6.0 | | 298.1±6.0 | | 302.9±6.4 | | | | 0.338 |  |  |
| Trans fatty acids | | | |  | |  | |  | | | |  |  |  |
| Median intake (g/d) | | 1.9 | | 2.5 | | 3.0 | | 3.8 | | | |  |  |  |
| Model 1 | | 297.4±6.1 | | 298.7±6 | | 303.9±6.1 | | 299.9±6 | | | | 0.461 |  |  |
| Model 2 | | 300.6±6.0 | | 299.2±5.8 | | 301.1±5.9 | | 299.1±5.8 | | | | 0.808 |  |  |
| Monounsaturated fatty acids | | | | | |  | |  | | | |  |  |  |
| Median intake (g/d) | | 25.0 | | 39.2 | | 32.7 | | 37.8 | | | |  |  |  |
| Model 1 | | 296.4±6.1 | | 304.5±6 | | 300±6 | | 299±6.1 | | | | 0.826 |  |  |
| Model 2 | | 298.3±6.0 | | 303.3±5.8 | | 298.8±5.8 | | 299.6±6.0 | | | | 0.973 |  |  |
| Polyunsaturated fatty acids | | | | | |  | |  | | | |  |  |  |
| Median intake (g/d) | | 8.0 | | 10.3 | | 12.6 | | 16.1 | | | |  |  |  |
| Model 1 | | 304.3±6.2 | | 301.6±6.1 | | 299.8±6.1 | | 294.2±6.2 | | | | 0.054 |  |  |
| Model 2 | | 301.4±6.0 | | 300±5.8 | | 301.3±5.8 | | 297.3±6.0 | | | | 0.631 |  |  |
| Values are means ± SEM | | | | | |  | |  | | | |  |  |  |
| Model 1 is adjusted for age, examination year and energy intake | | | | | | | | | | | | |  |  |
| Model 2 is adjusted for Model 1 and alcohol, smoking, BMI, years of education and physical activity | | | | | | | | | | | | |  |  |

| Supplemental Table 3. The mean serum concentrations of testosterone (nmol/L) in quartiles of energy-adjusted dietary fatty acid intakes among middle-aged and older men | | | | | | | |
| --- | --- | --- | --- | --- | --- | --- | --- |
|  | | Intake quartile | | | | |  |
|  | | 1 (n=636) | | 2 (n=637) | 3 (n=637) | 4 (n=636) | P-trend |
| Saturated fatty acids | | | | |  |  |  |
| Median intake (g/d) | | 38.0 | | 45.9 | 52.2 | 61.4 |  |
| Model 1 | | 20.2±0.3 | | 20.7±0.3 | 20.9±0.3 | 22.2±0.3 | <0.001 |
| Model 2 | | 20.6±0.3 | | 21.1±0.3 | 20.7±0.3 | 21.6±0.3 | 0.058 |
|  | |  | |  |  |  |  |
| Trans fatty acids | | | |  |  |  |  |
| Median intake (g/d) | | 1.9 | | 2.5 | 3.0 | 3.8 |  |
| Model 1 | | 20.9±0.3 | | 20.7±0.3 | 21.5±0.3 | 21.0±0.3 | 0.647 |
| Model 2 | | 21.2±0.3 | | 20.8±0.3 | 21.1±0.3 | 20.9±0.3 | 0.483 |
|  | |  | |  |  |  |  |
| Monounsaturated fatty acids | | | | |  |  |  |
| Median intake (g/d) | | 25.0 | | 39.2 | 32.7 | 37.8 |  |
| Model 1 | | 20.6±0.3 | | 21.7±0.3 | 21.1±0.3 | 20.7±0.3 | 0.827 |
| Model 2 | | 20.8±0.3 | | 21.5±0.3 | 21.0±0.3 | 20.8±0.3 | 0.693 |
|  | |  | |  |  |  |  |
| Polyunsaturated fatty acids | | | | |  |  |  |
| Median intake (g/d) | | 8.0 | | 10.3 | 12.6 | 16.1 |  |
| Model 1 | | 21.7±0.3 | | 21.1±0.3 | 21.0±0.3 | 20.3±0.3 | 0.008 |
| Model 2 | | 21.2±0.3 | | 20.9±0.3 | 21.2±0.3 | 20.7±0.3 | 0.532 |
| Values are means ± SEM | | | | |  |  |  |
| Model 1 is adjusted for age, examination year and energy intake | | | | | | | |
| Model 2 is adjusted for Model 1 and alcohol, smoking, BMI, years of education and physical activity | | | | | | | |
| Supplemental Table 4. The mean serum concentrations of SHBG (nmol/L) in quartiles of energy-adjusted dietary fatty acid intakes among middle-aged and older men | | | | | | | |
|  | Intake quartile | | | | | |  |
|  | 1 (n=636) | | 2 (n=637) | | 3 (n=637) | 4 (n=636) | P-trend |
| Saturated fatty acids | | | | |  |  |  |
| Median intake (g/d) | 38.0 | | 45.9 | | 52.2 | 61.4 |  |
| Model 1 | 38.9±0.7 | | 39.1±0.7 | | 40.3±0.7 | 43.3±0.7 | <0.001 |
| Model 2 | 39.7±0.7 | | 40.0±0.6 | | 40.1±0.6 | 41.9±0.7 | **0.029** |
|  |  | |  | |  |  |  |
| Trans fatty acids | | |  | |  |  |  |
| Median intake (g/d) | 1.9 | | 2.5 | | 3.0 | 3.8 |  |
| Model 1 | 40.4±0.7 | | 39.6±0.7 | | 41.5±0.7 | 40.2±0.7 | 0.860 |
| Model 2 | 41.1±0.6 | | 39.7±0.6 | | 40.7±0.6 | 40.1±0.6 | 0.486 |
|  |  | |  | |  |  |  |
| Monounsaturated fatty acids | | | | |  |  |  |
| Median intake (g/d) | 25.0 | | 39.2 | | 32.7 | 37.8 |  |
| Model 1 | 39.8±0.7 | | 42.1±0.7 | | 40.8±0.7 | 39.0±0.7 | 0.185 |
| Model 2 | 40.1±0.6 | | 41.7±0.6 | | 40.4±0.6 | 39.4±0.6 | 0.191 |
|  |  | |  | |  |  |  |
| Polyunsaturated fatty acids | | | | |  |  |  |
| Median intake (g/d) | 8.0 | | 10.3 | | 12.6 | 16.1 |  |
| Model 1 | 42.1±0.7 | | 40.7±0.7 | | 40.3±0.7 | 38.5±0.7 | 0.002 |
| Model 2 | 41.1±0.6 | | 40.2±0.6 | | 40.7±0.6 | 39.6±0.6 | 0.351 |
| Values are means ± SEM | | | | |  |  |  |
| Model 1 is adjusted for age, examination year and energy intake | | | | | | | |
| Model 2 is adjusted for Model 1 and alcohol, smoking, BMI, years of education and physical activity | | | | | | | |
